# Determination of Stable Co-Amorphous Drug–Drug Ratios from the Eutectic Behavior of Crystalline Physical Mixtures

**DOI:** 10.3390/pharmaceutics11120628

**Published:** 2019-11-24

**Authors:** Eric Ofosu Kissi, Keyoomars Khorami, Thomas Rades

**Affiliations:** 1 Department of Pharmacy, The Faculty of Mathematics and Natural Sciences, University of Oslo, P.O. Box 1068, Blindern, 0316 Oslo, Norway; e.o.kissi@farmasi.uio.no; 2 Department of Pharmacy, Faculty of Health and Medical Science, University of Copenhagen, Universitetsparken 2, DK-2100 Copenhagen, Denmark; keyoomars@gmail.com; 3 Faculty of Science and Engineering, Åbo Akademi University, Tykistökatu 6A, FI-20521 Turku, Finland

**Keywords:** physical stability, co-amorphous, eutectics, phase diagram

## Abstract

Co-amorphous drug–drug systems have been developed with the overall aim of improving the physical stability of two or more amorphous drugs. Co-amorphous systems often show good physical stability, and higher solubility and dissolution rates compared to their crystalline counterparts. The aim of this study is to determine if eutectic mixtures of two drugs can form stable co-amorphous systems. Three drug–drug mixtures, indomethacin–naproxen (IND−NAP), nifedipine–paracetamol (NIF−PAR), and paracetamol–celecoxib (PAR−CCX), were investigated for their eutectic and co-amorphization behavior as well as their physical stability in the co-amorphous form. The phase diagrams of the crystalline mixtures and the thermal behavior of the co-amorphous systems were analyzed by differential scanning calorimetry. The solid-state form and physical stability of the co-amorphous systems were analyzed using X-ray powder diffractometry during storage at room temperature at dry conditions. Initial eutectic screening using nifedipine (NIF), paracetamol (PAR), and celecoxib (CCX) indicated that IND−NAP, NIF−PAR, and PAR−CCX can form eutectic mixtures. Phase diagrams were then constructed using theoretical and experimental values. These systems, at different drug-to-drug ratios, were melted and cooled to form binary mixtures. Most mixtures were found to be co-amorphous systems, as they were amorphous and exhibited a single glass transition temperature. The stability study of the co-amorphous systems indicated differences in their physical stability. Comparing the phase diagrams with the physical stability of the co-amorphous mixtures, it was evident that the respective drug–drug ratio that forms the eutectic point also forms the most stable co-amorphous system. The eutectic behavior of drug–drug systems can thus be used to predict drug ratios that form the most stable co-amorphous systems.

## 1. Introduction

Poorly water-soluble crystalline drugs lead to low bioavailability and are a major challenge in the development of drug formulations for oral drug delivery [1]. Solubility can be improved on the molecular level, e.g., by salt formation, and on the colloidal level, e.g., by the use of lipid-based formulations [2]. For so-called “brick dust” molecules, i.e., poorly water-soluble drugs with medium polarity and (very) high melting points [3,4], improving the solubility on the particulate level is preferred. Improving solubility on the particulate level includes size reduction, nanosizing, the use of metastable polymorphic forms, and amorphization [2]. Amorphization is a deliberate process of converting ordered molecules, via either the thermodynamic or the kinetic pathway [4], into a disordered state with the overall aim of improving dissolution and solubility (supersaturation) of the drug in an aqueous phase [4]. The molecules in an amorphous solid form are arranged randomly and as a result exhibit higher free energy, enthalpy, and entropy than their respective crystalline forms [4,5]. 

A drawback to the use of amorphous drugs stems from the mobility of molecules in the glassy form [6,7]. Amorphous drugs have been found to be mobile (and thus physically unstable) at temperatures higher than the secondary glass transition temperature, the so-called *T_gβ_* [8]. Most amorphous drugs were found to have this *T_gβ_* at very low temperatures and this implies that when they are stored at room temperature (RT), they will revert from the amorphous form to their respective lower energy crystalline states [8,9]. Physical stability is therefore the main problem facing the development of amorphous drugs into solid dosage forms, such as capsules and tablets [10].

The main strategy involved in improving the physical stability of amorphous drugs is the formation of a glass solution [5,11,12,13]. Glass solutions can be classified into polymeric and non-polymeric systems [13,14]. Polymeric systems involve the dissolution of drug molecules into polymeric amorphous carriers, forming the well-known amorphous solid dispersion (ASD) [14,15,16]. ASDs stabilize amorphous drugs and additionally may increase their aqueous solubility, as well as the time the drug can maintain a supersaturated state in an aqueous medium [17]. Other stabilization processes that do not use polymers are termed non-polymeric glass solutions [13]. Co-amorphous systems are one group of these non-polymeric glass solutions and involve co-amorphization of two or more low molecular weight, initially crystalline, compounds. They form a single-phase amorphous system characterized by a single glass transition temperature (*T_g_*) [13]. Co-amorphous systems can be formed between a drug and a co-former using drug-excipient [18,19,20] and drug–drug combinations [21,22]. Crystalline drug–drug systems, which are not thermolabile, are interesting starting materials for co-amorphous systems as they may form eutectic mixtures. Eutectic mixtures, in this context, are drug–drug mixtures that at some drug–drug ratio are miscible in the molten state, usually at a temperature lower than the melting points of the individual drugs [4,5]. Eutectic mixtures can be used for combination therapy [23], improving solubility [24], and as starting materials for co-amorphous systems as they may reduce degradation [22].

In the development of co-amorphous drug–drug systems, selection of physically stable ratios of the two drugs is crucial. In view of this, this study focuses on determining if eutectic mixtures lead to physically stable co-amorphous systems and thus to use the eutectic behavior of drug–drug mixtures to predict drug–drug ratios that form the most stable co-amorphous systems. 

## 2. Materials and Methods 

### 2.1. Materials 

Naproxen (NAP, MW = 230.3 g/mol) was purchased from Sigma Aldrich (Steinheim, Germany). Indomethacin (IND, MW = 357.8 g/mol) and paracetamol (PAR, MW = 151.2 g/mol) were purchased from Fagron A/S (Copenhagen, Denmark). Celecoxib (CCX, MW = 381.4 g/mol) was purchased from Dr. Reddy’s (Hyderabad, India). Nifedipine (NIF, MW = 346.3 g/mol) was purchased from Hangzhou Dayangchem (Hangzhou, China). All materials were used as received.

### 2.2. Differential Scanning Calorimetry

Thermal analysis was performed using a differential scanning calorimetry (DSC) (Discovery DSC, TA Instruments Inc. New Castle, DE, USA). Samples of approx. 3–5 mg were crimped into Tzero aluminum pans and sealed with Tzero lids. For the determination of the eutectic points, crystalline physical drug–drug mixtures were equilibrated at 100 °C and further heated to 180 °C using a heating rate of 10 °C/min. For the determination of the *T_g_*, the samples were melted at 180 °C and then quickly cooled to 0 °C and kept isothermal for 5 min. The samples were then heated again, using the modulated temperature mode with an amplitude of 0.212 °C, a period of 40 s, and a heating rate of 5 °C/min.

### 2.3. X-Ray Powder Diffractometry

Solid-state forms were determined with an X’Pert PRO diffractometer (PANalytical, Almelo, Netherlands) using Cu Kα radiation (λ = 1.5406 Å) at 45 kV and 40 mA. Samples were placed on aluminum plates and measured over the angular range 5°–35° 2θ at a scanning speed of 0.058° 2θ/min and a step size of 0.026° 2θ. The diffractograms were analyzed using X’Pert HighScore Plus (version 2.2.4) software (PANalytical, Almelo, Netherlands).

### 2.4. Screening for Eutectic Mixtures

Binary crystalline physical mixtures of NIF, NAP, CCX, PAR, and IND were prepared at 1:1 molar ratios and placed on a hot plate whilst increasing the temperature gradually until a melt was observed. Combinations that melted below the melting temperature of the drug with the highest melting point and that were miscible in the molten form were considered for analysis by DSC and preparation of co-amorphous systems.

### 2.5. Determining Eutectic Points 

Crystalline physical mixtures (cPM) of the indomethacin–naproxen (IND−NAP), paracetamol–celecoxib (PAR−CCX), and nifedipine–paracetamol (NIF−PAR) binary systems were prepared by mixing and grinding using a mortar and pestle. The cPMs were melted in a DSC (see Section 2.2) and the temperature corresponding to the onset of the first melting event and the peak of the second melting signal were used to construct the phase diagrams [25]. The onset temperature of binary systems that showed a single melt endotherm and the corresponding drug–drug ratios were recorded as the eutectic point. All measurements were performed on independent triplicates. All drug–drug percentages expressed throughout this paper are in molar ratio (mol/mol, %).

### 2.6. Theoretical Values (Schröder–Van Laar Equation)

The eutectic behavior determined by DSC was compared with the predicted melting point (Tm) values calculated from the simplified version of the Schröder–Van Laar Equation [26,27].

(1) $$\ln\left(X\right)=\frac{\Delta H_{0}}{R}\left(\frac{1}{T_{0}}-\frac{1}{T}\right)$$

where Δ*H_0_* represents the heat of fusion (J·mol^−1^) and *T_0_* represents the melting point (in Kelvin) of one of the pure drugs in the mixture. *T* is the melting point of the binary mixture at a specific mole fraction, *X*, and *R* is the gas constant (8.314 J·K^−1^·mol^−1^).

### 2.7. Preparation of Co-Amorphous Systems

Co-amorphous systems were prepared from the cPM by melting at 5 °C above the melting point of the drug with the highest melting point. The molten drugs were stirred with a spatula to avoid the formation of two amorphous phases and were quickly cooled using an ice gel pack. The formed glass was then gently milled into powder, using a mortar and pestle, for further characterization and physical stability studies. 

### 2.8. Physical Stability Studies

Physical stability studies were performed using the co-amorphous PAR−CCX, NIF−PAR, IND−NAP samples, and their neat amorphous starting materials. The samples were stored at 0% relative humidity (RH) using phosphorus pentoxide (P_2_O_5_) at RT. All the neat amorphous drugs were analyzed daily until they recrystallized. For co-amorphous IND−NAP samples, XRPD analyses were performed on a daily basis for one week, then weekly until all triplicate samples recrystallized. For co-amorphous PAR−CCX samples, XRPD analyses were performed weekly for one month, then monthly until all triplicate samples crystallized. For NIF−PAR, XRPD analyses were made daily until all samples crystallized. All measurements were performed on independent triplicates.

## 3. Results

A eutectic system is a mixture of two compounds which do not interact to form a new chemical compound, but at a specific ratio, i.e., the eutectic point, exhibit a single Tm which is lower than the Tm of the individual components [25,28]. Eutectic mixtures are miscible in the molten form and when amorphized may improve the physical stability and solubility of the resulting co-amorphous system [22].

### 3.1. Screening for Eutectic Mixtures

**Figure 1:**
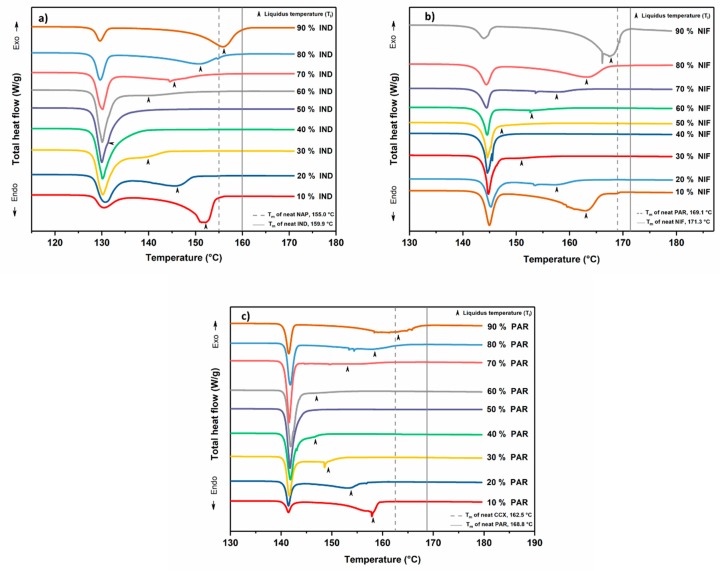
Differential scanning calorimetry (DSC) thermograms of the crystalline physical mixtures of (**a**) indomethacin–naproxen (IND−NAP), (**b**) nifedipine–paracetamol (NIF−PAR), and (**c**) paracetamol–celecoxib (PAR−CCX). The black arrows indicate the liquidus temperatures.

Initial screening of binary crystalline mixtures of NAP, IND, CCX, PAR, and NIF showed that at the 1:1 molar ratio, PAR−CCX, NIF−PAR, and IND−NAP were molten and miscible at relatively low temperatures and are thus candidates that may form a eutectic mixture. Previous studies by Beyer et al. and Löbmann et al. have indeed shown that IND−NAP can form eutectic mixtures and has a eutectic point at around 55% to 60% NAP [21,22]. After the initial screening, DSC was used to determine the phase diagrams and the eutectic points of PAR−CCX, NIF−PAR, and IND−NAP samples. The various drug–drug combinations were prepared in steps of 10% molar ratio (mol/mol, %). The thermograms in Figure 1 show a melting endotherm of which the onset temperatures do not change with increasing drug concentration. This temperature is the solidus temperature below which the mixture is in its solid state (crystalline mixture). For the second melting endotherm, however, the onset temperatures shift towards or away from the solidus temperature depending on drug molar ratios. This is a clear indication that above a certain temperature, i.e., the liquidus temperature, the drug–drug mixtures will be in the molten state. The observed melting temperatures for the drug–drug mixtures were recorded and the phase diagrams were constructed.

### 3.2. Phase Diagrams 

**Figure 2:**
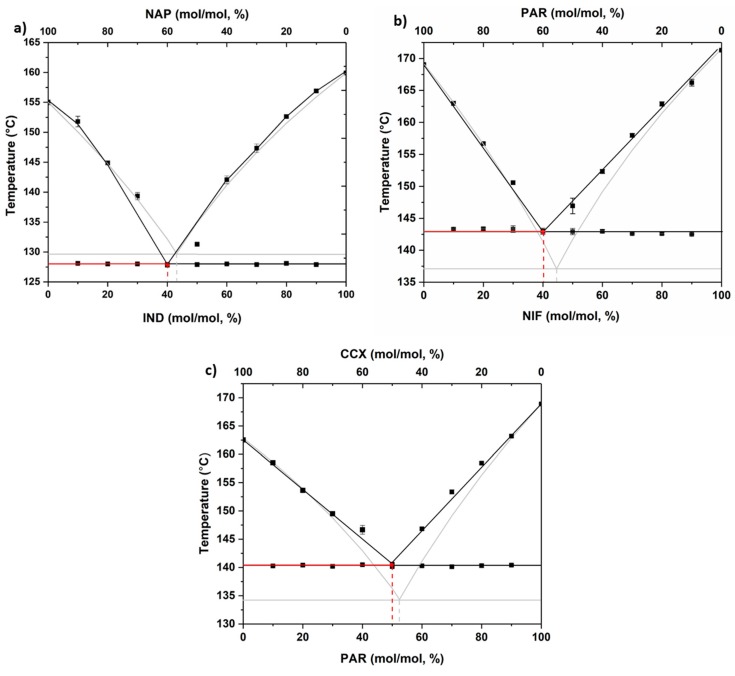
Phase diagrams of (**a**) IND−NAP samples, (**b**) NIF−PAR samples, and (**c**) PAR−CCX samples using experimentally determined data (black square) and theoretical data using the Schröder–Van Laar equation (grey line).

The experimentally determined solidus and liquidus temperatures were plotted against the drug concentration to construct the phase diagrams. In addition, theoretical values, based on the Schröder–Van Laar equation (see Section 2.6), were added and are shown in Figure 2. The figures are typical of eutectic systems and show the liquidus and solidus curves and the experimental and theoretical points at which these curves meet; i.e., the eutectic points [29,30,31]. The eutectic ratios and their corresponding temperatures (*T_e_*), both experimentally and theoretically determined for all analyzed drug–drug mixtures, are shown in Table 1. The theoretical eutectic points did not differ by more than 10% from the experimentally determined values. This shows that these systems can form eutectics and are suitable candidates for the investigation of the importance of the eutectic mixture in determining the most stable co-amorphous systems.

### 3.3. Preparation and Characterization of the Co-amorphous Systems 

**Figure 3:**
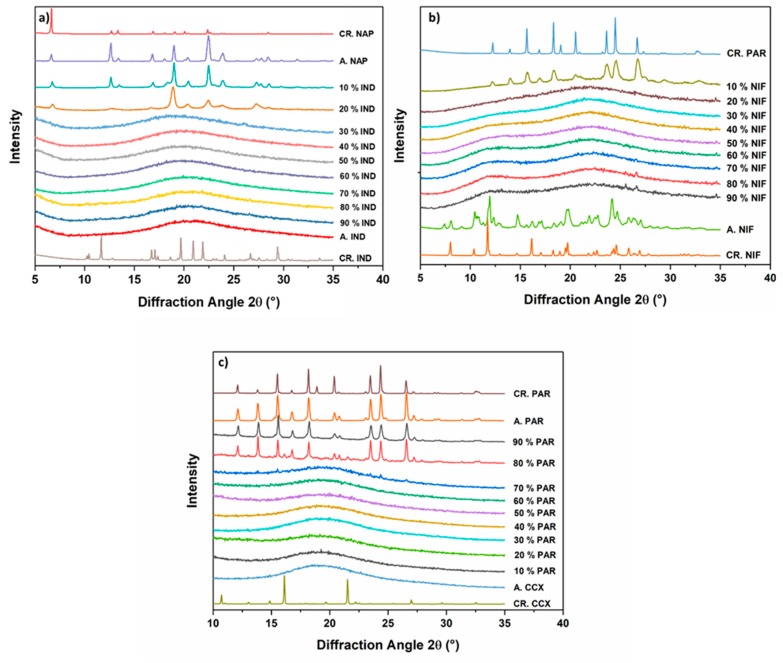
Diffractograms of (**a**) IND-NAP samples, (**b**) NIF-PAR samples, and (**c**) PAR-CCX samples after melt quenching. Included are the diffractograms of starting materials (CR. NAP, CR. IND, CR. PAR, CR. NIF, and CR. CCX) and the single drugs after melting and cooling (A.NAP, A. IND, A. PAR, A. NIF, and A. CCX).

Fresh cPM were prepared at different molar ratios, and the thermodynamic pathway (melting and rapid cooling) was used to produce co-amorphous systems. Rapid cooling, using ice gel packs, was used since varying the cooling rate can have an effect on the physical stability of the samples [21]. Solid-state characterization was performed with XRPD and the results are shown in Figure 3. Figure 3a shows the diffractograms of the co-amorphous IND−NAP samples. NAP could not be amorphized on its own and the diffractogram shows recrystallization after fast cooling from the melt. IND−NAP samples with a low concentration of IND, i.e., drug–drug ratios containing between 10% and 20% IND, did show diffraction peaks of crystalline NAP after melt quenching. From Figure 3b,c, diffractograms of samples containing 10% NIF and 80%–90% PAR, respectively, did show peaks of PAR. 

**Figure 4:**
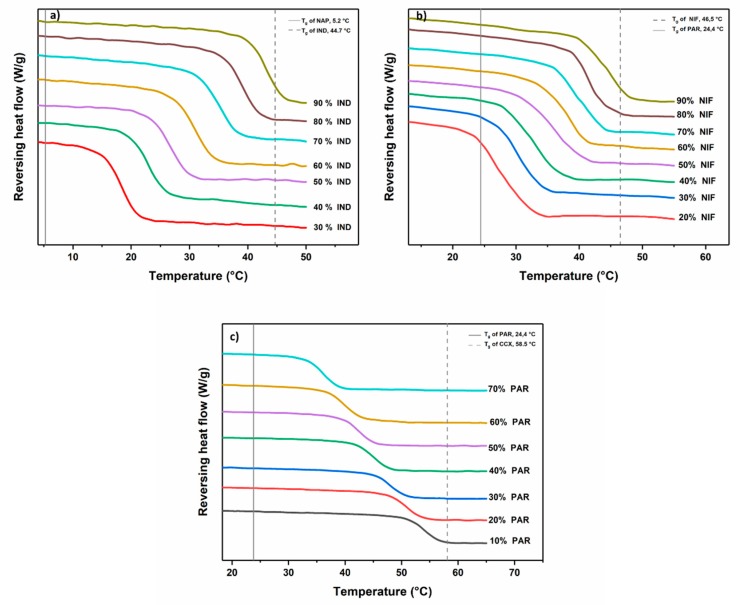
DSC thermograms showing the change in *T_g_*s of the various co-amorphous (**a**) IND-NAP samples, (**b**) PAR-CCX samples, and (**c**) NIF-PAR samples.

In contrast, the remaining drug–drug mixtures did show a halo without distinct peaks in the XRPD diffractograms. A halo pattern in a diffractogram is a characteristic property of amorphous solids. To confirm if a co-amorphous system has been formed, the drug–drug mixtures that showed a halo pattern in the diffractograms were subjected to DSC analysis and the results are shown in Figure 4. These drug–drug mixtures exhibited a single *T_g_* and for IND-NAP (Figure 4a), the *T_g_* ranges from 18.2–42.9 °C (with increasing IND concentration). The *T_g_* range for NIF−PAR samples and PAR−CCX samples (Figure 4b,c), are 28.0–44.3 °C (with increasing NIF concentration) and 36.3–54.7 °C (with increasing CCX concentration), respectively. Characterization using DSC and XRPD confirms that IND−NAP samples containing 30%–90% IND, NIF−PAR samples containing 20%–90% NIF, and PAR−CCX samples containing 10%–70% PAR respectively were co-amorphized successfully. 

### 3.4. Physical Stability of Co-Amorphous Systems

**Figure 5:**
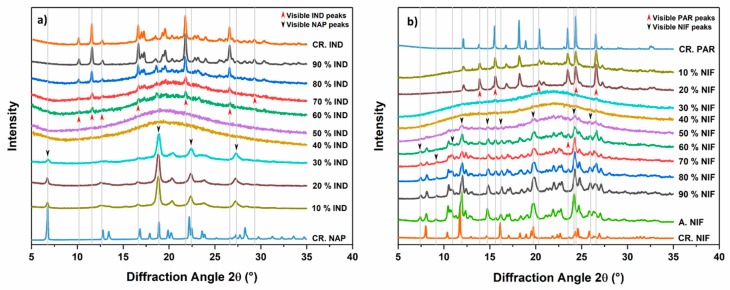
Diffractograms of (**a**) IND−NAP samples and (**b**) NIF−PAR samples after storage at 0% relative humidity (RH) and at RT for 14 days. Included in the diffractograms are crystalline starting materials (CR. IND, CR. NAP, and CR. NIF) and the diffractogram for NIF a day after melting and cooling (A. NIF).

A physical stability analysis was performed to determine the tendency towards recrystallization of the co-amorphous systems when stored dry at RT. Figure 5 shows the diffractograms of the various co-amorphous systems. After storing co-amorphous IND−NAP samples for 14 days, all diffractograms (Figure 5a) showed diffraction peaks except for those samples containing 40% and 50% IND. Diffractograms of co-amorphous NIF−PAR samples after dry storage for 14 days (Figure 5b), revealed that samples containing 30% and 40% NIF showed an amorphous halo and therefore maintained their amorphous form. 

A general trend that can be observed in the diffractograms in Figure 5 is the recrystallization pattern of the various co-amorphous systems, above or below the drug concentrations that showed a halo. It can be seen that these co-amorphous systems recrystallized into one of the starting materials, i.e., no diffraction peaks of the other starting material could be detected. This trend of recrystallization was also found in our previous co-amorphous drug-amino acid studies. In that study, we found that when co-amorphous systems recrystallize into one of the starting materials, this material has to be regarded as the excess component in the system [32]. 

### 3.5. Eutectics and Physical Stability

**Figure 6:**
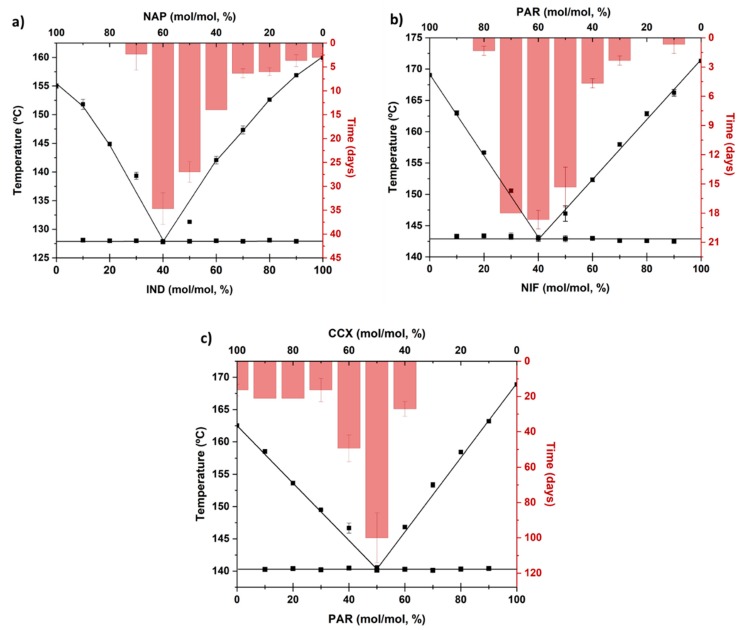
Time for the onset of crystallization (bar chart) for various co-amorphous (**a**) IND−NAP samples, (**b**) NIF−PAR samples, and (**c**) PAR−CCX samples, and a comparison to their respective phase diagram.

IND−NAP, NIF−PAR, and PAR−CCX form eutectic mixtures and can be cooled quickly from the molten state to form a co-amorphous system. The co-amorphous systems, however, showed differences in their physical stability (time taken for the onset of crystallization). Here we relate the physical stability to the eutectic behavior. The physical stability data of the different co-amorphous systems have been superimposed on their respective phase diagrams and the results are shown in Figure 6. IND−NAP samples (Figure 6a) containing 10%–20% IND recrystallized immediately after preparation. In contrast, co-amorphous IND-NAP sample formed from the eutectic ratio of the two drugs, i.e., 40% IND and 60% NAP, are physically stable between 31 and 38 days, which for IND is a more than tenfold increase compared to the neat amorphous drug. Increasing the concentration of IND above the ratio at the eutectic point reduces the physical stability gradually towards that of neat amorphous IND. 

The onset of crystallization of NIF−PAR samples is shown in Figure 6b. Neither NIF nor PAR are physically stable and will crystallize within one day of storage at 0% RH and at RT. Therefore, the physical stability of the co-amorphous forms was determined daily for 20 days. NIF−PAR samples containing from 30%–50% NIF were stable between 13 and 20 days, and the most stable sample was found to be the NIF−PAR samples that contained 40% NIF (recrystallized between 17 and 20 days), i.e., at the drug–drug ratio that corresponded to the eutectic point mixture.

The onset of crystallization of PAR−CCX samples is shown in Figure 6c. The physical stability for samples with a PAR concentration of 10%–30% is similar to that of neat amorphous CCX, which is stable for less than 20 days. When increasing the PAR concentration to up to 40%, the physical stability increases considerably to over 49 days. At the eutectic point (PAR−CCX sample containing 50% PAR), the sample exhibits higher physical stability compared to the other PAR−CCX samples studied, and recrystallizes between 86–114 days. However, further increasing the concentration of PAR to 60% decreases the physical stability to below 35 days. Samples with a high concentration of PAR recrystallized almost immediately after preparation. 

In summary, this study shows that the physical stability of co-amorphous systems can be deduced from the eutectic behaviors of their respective crystalline mixtures. The most stable co-amorphous ratios were found at ratios where the crystalline mixtures of the drugs had their respective eutectic points. The observed trend in using eutectics to predict physical stability should be extended to systems that have their eutectic points at low or high concentrations since the eutectic ratios of the studied co-amorphous samples are close to 1:1 molar ratios. 

## 4. Conclusions

In this study, we have studied three eutectic drug–drug mixtures, IND−NAP, NIF−PAR, and PAR−CCX, and confirmed that the respective co-amorphous drug–drug mixtures are most stable at a ratio corresponding to the eutectic point mixture of the respective crystalline drug–drug mixtures. The eutectic behavior of drug–drug mixtures can thus serve as a screening tool for finding optimally stable co-amorphous systems, and in the selection of physically stable co-amorphous drug–drug ratios, the composition making up the eutectic point may be selected for further development.

## Figures and Tables

**Table 1:** Experimental and theoretical eutectic point and *T_e_* values for IND−NAP, NIF−PAR, and PAR−CCX.

Eutectic point and *T_e_*	IND−NAP	NIF−PAR	PAR−CCX
Experimental eutectic point mixture (mol/mol, %)	40:60	40:60	50:50
Experimental *T_e_* (°C)	127.9	143.0	140.6
Theoretical eutectic point mixture (mol/mol, %)	43:57	44:56	52:48
Theoretical *T_e_* (°C)	129.7	137.2	134.2

*T_e_*: eutectic point temperature.
